# An Optimal Choice of Cognitive Diagnostic Model for Second Language Listening Comprehension Test

**DOI:** 10.3389/fpsyg.2021.608320

**Published:** 2021-04-16

**Authors:** Yanyun Dong, Xiaomei Ma, Chuang Wang, Xuliang Gao

**Affiliations:** ^1^School of Foreign Studies, Xi'an Jiaotong University, Xi'an, China; ^2^Faculty of Education, University of Macau, Taipa, China; ^3^School of Psychology, Guizhou Normal University, Guiyang, China

**Keywords:** cognitive diagnostic model, L2 listening subskills, model selection, mixed-CDMs, inter-attribute relationship

## Abstract

Cognitive diagnostic models (CDMs) show great promise in language assessment for providing rich diagnostic information. The lack of a full understanding of second language (L2) listening subskills made model selection difficult. In search of optimal CDM(s) that could provide a better understanding of L2 listening subskills and facilitate accurate classification, this study carried a two-layer model selection. At the test level, A-CDM, LLM, and R-RUM had an acceptable and comparable model fit, suggesting mixed inter-attribute relationships of L2 listening subskills. At the item level, Mixed-CDMs were selected and confirmed the existence of mixed relationships. Mixed-CDMs had better model and person fit than G-DNIA. In addition to statistical approaches, the content analysis provided theoretical evidence to confirm and amend the item-level CDMs. It was found that semantic completeness pertaining to the attributes and item features may influence the attribute relationships. Inexplicable attribute conflicts could be a signal of suboptimal model choice. Sample size and the number of multi-attribute items should be taken into account in L2 listening cognitive diagnostic modeling studies. This study provides useful insights into the model selection and the underlying cognitive process for L2 listening tests.

## Introduction

Cognitive diagnosis models (CDMs), also known as diagnostic classification models (Rupp et al., 2010), show great promise for producing rich diagnostic information about students' strengths and weaknesses on a set of finer-grained attributes (Rupp and Templin, 2008). Although a wide array of CDMs have been developed and widely used in language assessment, listening comprehension receives little attention compared with other language skills in the second language (L2) research.

Previous studies, though sparse, have shown the feasibility of applying CDMs to L2 listening comprehension tests and demonstrated the potential usefulness of cognitive diagnostic approaches (CDAs) to understanding various subskills (Buck and Tatsuoka, 1998; Lee and Sawaki, 2009a; Sawaki et al., 2009; Aryadoust, 2011; Meng, 2013; Yi, 2017). However, it is not very clear which CDM should be used for L2 listening comprehension tests. Some studies used non-compensatory models (e.g., Buck and Tatsuoka, 1998; Sawaki et al., 2009), whereas others concluded that compensatory and non-compensatory models produced striking similar diagnostic results for listening comprehension tests (Lee and Sawaki, 2009a). Still, others used G-DINA, which allows compensatory and non-compensatory inter-attribute relationships within the same test (Meng, 2013).

Selecting the right CDM(s) for a given test is of critical importance because model selection affects the diagnostic classification of examinees (Lee and Sawaki, 2009a) and thus influences the accuracy of diagnostic feedback. Providing learners with accurate diagnostic feedback to guide their remedial learning is the ultimate goal of CDA (Lee and Sawaki, 2009b). Wrong CDM(s) for a given dataset will lead to the wrong classification of examinees and misleading feedback. In addition, CDMs can provide information on the underlying inter-attribute relationships (Yi, 2017), that is, whether a compensatory or non-compensatory inter-attribute relationship can produce correct answers. Wrong CDM(s) for given data will generate a wrong interpretation of inter-attribute relationships and skill mastery status. The sample size is also one concern. Reduced models require a smaller sample size to be estimated accurately, although the saturated model can provide better model-data fit at test level compared with other reduced models; appropriate reduced models can provide better classification rates than saturated models, particularly when the sample size is small (Rojas et al., 2012). Non-parametric models can accommodate small samples, but they could not commonly use model-data fit indices of parametric methods to compare with parametric models. This is because it will be difficult to tell whether parametric or non-parametric models are better for the given data without parameter estimation. Therefore, it is inconvenient to make a model selection with other commonly used CDMs (Kang et al., 2019). Non-parametric models need prior knowledge of the inter-attribute relationships (compensatory or non-compensatory) of a given skill to decide whether a compensatory or non-compensatory model should be selected (Chiu and Douglas, 2013). In sum, inappropriate CDMs lead to inaccurate diagnostic classification, wrong interpretation of skill mastery status, and misunderstanding of inter-skill relationships. Few empirical studies, however, have examined the model comparison and selection for L2 listening comprehension tests, and little is known about L2 listening inter-skill relationships.

## Theoretical Framework and Literature Review

### Listening Comprehension Skills

Listening comprehension is the least-researched skill among the four skills of reading, listening, writing, and speaking (Bae and Bachman, 1998; Field, 2013). Although some researchers assert that listening comprehension is a more integrated skill (Levine and Revers, 1988) and not empirically multi-divisible (Oller, 1983; Wagner, 2004), most researchers agree that listening comprehension involves multiple subskills (Rivers, 1966; Carroll, 1972; Clark and Clark, 1977; Bae and Bachman, 1998; Buck and Tatsuoka, 1998; Song, 2008; Rost, 2011; Field, 2013). Munby (1978) and Richard (1983) presented a complete taxonomy of subskills, but the inter-subskill relationships are hard to explain. In contrast, Aitken (1978) provided a succinct taxonomy from the communicative approach by recognizing major listening subskills. Weir (1993) is along a similar line of defining important listening subskills only. The idea of major listening subskills benefits listening comprehension test development and studies in terms of the construct validity of these tests, especially studies with CDA (Buck and Tatsuoka, 1998; Lee and Sawaki, 2009a; Sawaki et al., 2009; Yi, 2017).

In addition to the widely discussed issue of the divisibility of listening comprehension (e.g., Bae and Bachman, 1998), listening subskill relationships were manifested by factor analysis of test-takers' responses (Liao, 2007; Shin, 2008; Song, 2008). Goh and Aryadoust (2015), however, argued that the structure of interactive and interdependent listening subskills was much more complicated than what factor analysis could represent. This argument echoed the view of Buck (2001) that “various types of knowledge involved in understanding language are not applied in any fixed order—they can be used in any order, or even simultaneously, and they are all capable of interacting and influencing each other” (p. 3). It is worth noting that the assertion of Buck (2001) shows the interactive and varied subskill relationships of listening, which also implies that the relationships among listening subskills are not yet clear.

### Model Selection for L2 Listening Comprehension Tests

Each CDM has unique assumptions about the latent attribute relationships (e.g., compensatory or non-compensatory). Under a compensatory CDM, successfully mastering only one or some of the required attributes may compensate for the non-mastery of others. In contrast, under a non-compensatory CDM, an item can be correctly answered only if all the required attributes have been mastered. If the assumption of a CDM does not match the latent attribute relationships of given data, the CDM is improper for the test and cannot offer accurate classification and diagnostic feedback. Test-level model selection is based on clear inter-attribute relationships. If the relationships are not clear, selecting the most appropriate CDM(s) will be a challenge.

In the literature, both compensatory and non-compensatory CDMs were applied to the L2 listening comprehension test. Buck and Tatsuoka (1998) applied the rule-space model to an L2 listening comprehension test. Aryadoust (2011) used the fusion model (FM) to a version of the International English Language Testing System listening comprehension test, and Sawaki et al. (2009) also used FM to the Test of English as a Foreign Language iBT listening comprehension items. The rule-space model and FM are both non-compensatory models. Meng (2013) applied G-DINA to an L2 listening comprehension test. G-DINA accommodates both compensatory and non-compensatory inter-attribute relationships. In addition, Lee and Sawaki (2009a) concluded that compensatory and non-compensatory models produced strikingly comparable diagnostic results. Yi (2017), however, argued that a compensatory model (C-RUM) was the best to interpret the listening subskill relationships. As both compensatory and non-compensatory CDMs were applied to L2 listening comprehension tests in the literature, CDM selection for L2 listening comprehension tests is still inconclusive and deserves further exploration.

### Cognitive Diagnostic Models

*G-DINA* model (de la Torre, 2011) is a saturated model and considers all possible interaction effects among required subskills for an item. It classifies examinees into $2^{K_{j}^{*}}$ latent groups based on mastery of required skills for each item. $K_{j}^{*}$ is the number of attributes required for item *j*. $\alpha_{lj}^{*}$ is the reduced attribute vector whose elements are the required attributes for item *j*. If one item needs two attributes, these two attributes lead to four latent groups: those who mastered both attributes, one of the attributes, or none of the attributes. Its item response function based on $P\left(a_{lj}^{*}\right)$ is as follows:

$$\begin{array}{r} P\left(\alpha_{lj}^{*}\right)=\delta_{j0}+\overset{K_{j}^{*}}{\underset{k=1}{\sum }}\delta_{jk}\alpha_{lk}+\overset{K_{j}^{*}}{\underset{k^{\prime }=k+1}{\sum }}\overset{K_{j}^{*}-1}{\underset{k=1}{\sum }}\delta_{jkk^{\prime }}\alpha_{lk}\alpha_{lk^{\prime }}...\\ +\delta_{j12...K_{j}^{*}}\overset{K_{j}^{*}}{\underset{k=1}{\prod }}\alpha_{lk} \end{array}$$

δ_*j*0_ is the intercept for item *j*, representing the baseline probability of a correct response when none of the required subskills is present. δ_*jk*_ is the main effect of mastering a single-skill α_*k*_, representing the change in the probability of a right answer (PRA) as a result of mastering a single skill. *δ_jkk′_* is the first-order interaction effect due to mastering both α_*k*_ and $\alpha_{k^{\prime }}$. $\delta_{j12...K_{j}^{*}}$ is the highest order interaction effect due to mastering all the required subskills up to $K_{j}^{*}$ (de la Torre, 2011). G-DINA is often used as the benchmark model when the true model is not known (Chen et al., 2013; Li et al., 2016).

Under the framework of G-DINA, there are some special cases called the reduced models: DINA, DINO, A-CDM, R-RUM, and LLM, which are used in this study and introduced as follows:

The *DINA* (deterministic inputs, noisy, “and” gate; Juncker and Sijtsma, 2001) model is a special case by setting all the parameters of G-DINA, except δ_*j*0_ and $\delta_{j12...K_{j}^{*}}$, to zero. Thus, DINA is a non-compensatory model, assuming that examinees have to master all the required skills simultaneously to choose the correct answer.

If setting the values of all the main and interaction effect parameters of G-DINA to be the same or, in other words, the main and interaction effects are identical to each other, then *DINO* (deterministic input, noisy, “or” gate; Templin and Heson, 2006) is obtained. DINO is the compensatory counterpart of DINA, assuming that examinees can have the same PRA whether they master one required subskill or all.

*A-CDM* (additive CDM; de la Torre, 2011) can be obtained when all the interaction effects in G-DINA are set to zero while keeping the compensatory property. This model indicates that mastering one subskill increases the PRA on an item, and its contribution is independent of the other subskills.

*R-RUM* (Reduced Reparameterized Unified Model; Hartz, 2002) is a non-compensatory model with a log link, setting the interaction terms to zero. It is considered a non-compensatory counterpart of A-CDM (Hartz, 2002).

If using a logit link, setting the interaction terms to zero and keeping the compensatory property, *LLM* (linear logistic model; Hagenaars, 1990, 1993; Maris, 1999) is obtained. Similar to A-CDM, it also assumes that the mastery of one subskill will increase the PRA to the item.

There is another group of models, named non-parametric models, which do not require a sample size. Because there is no parameter estimation in non-parametric models, it is not possible to make model comparisons with parametric models based on common fit indices (Kang et al., 2019). Prior knowledge of attribute relationships is required for non-parametric model selection, but unknown attribute relationships in this study make it more difficult to compare with parametric CDMs. As a result, non-parametric models were not be used in this study.

The following research questions guided this study.

1. Which model is the best for the second language listening comprehension test at the test level when the sample size is small?

2. Which model is the best for the second language listening comprehension test at the item level when the sample size is small?

3. What are the inter-attribute/subskill relationships of second language listening comprehension?

## Methods

### Participants

Participants were 500 freshmen (149 females and 351 males) conveniently sampled from four universities in the northwest region of China. They all majored in science and technology and aged between 17 and 20 years old. This sample size is of practical importance, although it was considered small in previous simulation studies of CDMs (de la Torre and Lee, 2013; Ma et al., 2016).

### Instrument

#### L2 Listening Diagnostic Assessment (L2LDA)

*L2LDA* is part of the *English as Foreign Language Listening Diagnostic Test* in the *PELDiaG system (Personalized English Learning Diagnosis and Guidance system)* designed for the diagnostic purpose (Meng, 2013; Ma and Meng, 2014; Du and Ma, 2018). The original test in the *PELDiaG system* has two types of items: multiple-choice items and sentence-dictation items. Only the 19 multiple-choice items, which are dichotomously scored, were used in this study. Sentence-dictation items were not dichotomous and scored holistically within a score range from 0 to 3.5 points. For the reason of convenience, these items were excluded in this study. *L2LDA* has four sections of short conversations, a long conversation, short passages, and a video clip. The topics of it cover campus life, social life, and common scientific knowledge, which largely reduced the possibility of bias caused by topic preference. The participants' total scores of the *L2LDA* followed a normal distribution with a mean score of 11.50 (out of a total score of 19) and a standard deviation of 4.05.

#### Q-Matrix

A Q-matrix, a critical input of CDMs, specifies the relationship between attributes and test items. Because sentence-dictations (tapping into the attribute of Short-term Memory and Note Taking) were excluded from this study, the attributes of Short-term Memory and Note Taking were accordingly canceled from the original Q-matrix (Dong et al., 2020), and then, six attributes were retained in the Q-matrix (Table 1) for this study.

**Table 1:** Configuration of Q-matrix.

	**Attributes**							**Attributes**					
	**A1**	**A2**	**A3**	**A4**	**A5**	**A6**		**A1**	**A2**	**A3**	**A4**	**A5**	**A6**
Item 1	1	0	0	0	0	0	Item 11	0	1	0	0	0	1
Item 2	1	0	0	0	0	0	Item 12	0	0	0	0	0	1
Item 3	0	1	0	1	0	0	Item 13	0	0	0	1	1	0
Item 4	0	1	0	1	0	0	Item 14	0	0	0	0	0	1
Item 5	0	1	0	0	0	0	Item 15	0	0	0	0	0	1
Item 6	0	0	1	0	0	0	Item 16	0	0	1	0	1	0
Item 7	0	0	0	1	0	0	Item 17	0	0	0	0	0	1
Item 8	0	0	1	0	0	0	Item 18	0	0	0	0	1	0
Item 9	0	0	1	0	0	0	Item 19	0	0	0	1	1	0
Item 10	0	0	0	1	1	0							

The six subskills/attributes for this study in relation to the existing listening skill taxonomies are presented in Table 2. Their definitions in accord with the ones identified by Meng (2013) are the following:

A1: Sound Discrimination: Recognizing special phonological and prosodic information, such as liaison and assimilation, stress and weak forms, intonation.
A2: Less Frequent Vocabulary and Expressions: Understanding less frequent words, oral expressions, and slangs.
A3: Difficult Structures: Difficult sentence structure and grammatical functions such as subjunctive mood, inversion, and negation.
A4: Facts and Details: Understanding detailed expressions of time, places, and relationships.
A5: Main Idea: Recognizing and summarizing main ideas and major points.
A6: Situational Context and Cultural Background Inferences: Obtaining motivations, purposes, reasons, and interactive functions by inferring from the context, implied expressions, and cultural background.

**Table 2:** Listening attributes/subskills and relationship with existing listening skill taxonomies.

**Listening attributes/subskills and definitions**	**Listening Cognitive Ability of China Standards of English (He and Chen, 2017)**	**Field's (2009)**	**Aitken's (1978)**	**Sawaki et al.'s (2009)**
A1: Sound discrimination	Identify/retrieve	Input decoding	Understand prosodic patterns	
A2: Less frequent vocabulary and expressions	Identify/retrieve	Lexical search	Understand vocabulary	Understand vocabulary
A3: Difficult structures	Identify/retrieve/analyze	Parsing	Understand syntactic patterns	
A4: Facts and details	Identify/retrieve/analyze	Meaning construction		Understand important information
A5: Main idea	Analyze/summarize/create	Meaning construction; Discourse construction		Understand overall topic/gist;
A6: Situational context and cultural background inference	Analyze/summarize/ create/evaluate	Meaning construction; Discourse construction	Identify speaker's purpose, attitudes, views, and intentions; Making inferences;	Making inferences
			Identify rhetorical devices.	Understand the structure (rhetorical, discourse).

*The attributes and definitions stem from the study of Meng (2013, p. 78, p. 95)*.

### Data Analytical Procedure

Three major procedures were followed: (a) Model selection; (b) Empirical comparisons between G-DINA, the most comparable CDM with G-DINA at the test level and the selected CDM(s) at the item level in terms of psychometric characteristics; and (c) Content analysis that is required to confirm or amend the selected item-level models.

### CDM Selection

The R GDINA package (Ma and de la Torre, 2016) was used for model estimation and selection. G-DINA was used as the baseline model and was compared with the other reduced models: DINA, DINO, R-RUM, A-CDM, and LLM. The absolute model fit and the relative model fit were used to compare the models. The absolute fit indices were calculated based on the residuals between the observed and predicted Fisher-transformed correlations of item pairs [Max.z(*r*)] and between the observed and predicted log-odds ratios of item pairs [Max.z(*l*)]. The least critical *p*-value was 1% (Chen et al., 2013). The second absolute fit index is M_2_ (Maydeu-Olivares and Joe, 2006), which is a limited-information fit statistic, and 0.05 is the critical *p*-value. The third is the root mean squared error approximation (RMSEA), which reflects the discrepancy between the predicted and the observed tetrachoric correlation for all pairs of items. RMSEA value of 0.05 was used to assess model fit (Henson and Templin, 2007). The fourth is the standardized root mean square residual (SRMSR), which is the square root of the sum of the squared differences of the observed correlation and the model implied correlation of all item pairs. SRMSR below 0.05 indicates a good absolute fit (Maydeu-Olivares, 2013). All the above are the absolute fit indices provided by the G-DINA package, and they serve as initial screening tools (Yi, 2017). Subsequently, the relative fit indices play a more critical role in narrowing down the scope of CDMs.−2Log-likelihood(-2LL), Akaike Information Criterion (AIC), and Bayesian Information Criterion (BIC) values are relative fit indices. Because−2LL always selects the saturated model and BIC imposes the biggest penalty (Lei and Li, 2014), AIC was used to compare G-DINA with the other models in this study.

In addition to holistically selecting models at the test level, CDMs were also selected at the item level. Language tests commonly include two kinds of items in terms of how many attributes are measured in each item: single-attribute item and multi-attribute item. The Wald test was used to select the most appropriate reduced CDMs for multi-attribute items (de la Torre and Lee, 2013; Ma et al., 2016). For single-attribute items, no distinction can be made between general and reduced CDMs, and then G-DINA was used. In *L2LDA*, Items 3, 4, 10, 11, 13, 16, and 19 are two-attribute items, but all other items were single-attribute. The reduced CDM whose *p*-value of Wald statistics was > 0.05 was accepted. When more than one reduced model was acceptable, the model with the largest *p*-value stayed. If DINA or DINO was one of the retained models, the DINA or DINO models were preferred over the other three models because of their simplicity. If all reduced models were rejected for an item, G-DINA was chosen. Thus, Mixed-CDMs were formed for this test.

### CDM Comparison

Accordingly, after the selection of Mixed-CDMs, psychometric properties (i.e., absolute fit, absolute item fit, relative fit, person fit, and attributes classification reliability) under G-DINA as a baseline, the most comparable CDM with G-DINA at the test level, Mixed-CDMs, and G-DINA were compared. The absolute fit and relative fit used the same indices as in the test-level model selection. The *l*_*z*_index was used for person fit. The *l*_*z*_index is standardized, so a value of 0.0 reflects an ideally perfect typical response string (Drasgow et al., 1985). *l*_*z*_ > 2.0 indicates over-fitness, whereas *l*_*z*_below −2.0 indicates poor fit. Test-level and attribute-level classification accuracy indices estimated from the GDINA function followed the approaches in Iaconangelo (2017) and Wang et al. (2015).

Then item parameters under G-DINA and the selected models were examined. Item parameter statistics can inform the inter-attribute relationships, which give evidence to inference interpretability analysis. Next, content analysis of the two-attribute items was carried out to justify or modify the results of model selection. Inter-attribute relationships can be informed by item parameter estimations, which are different under different CDMs. Content analysis was to examine whether the inter-attribute relationships manifested by the selected model were reasonable.

## Results

### Model Selection at Test Level

Table 3 summarizes the model fit results and the numbers of parameters of the six models. G-DINA, A-CDM, LLM, and R-RUM could be accepted with the significant levels of both Max.z(*r*) and Max.z(*l*) being much higher than 1% (the least critical *p*-value), whereas DINA and DINO could not be accepted with the significant levels of Max.z(*r*) being lower than 1%, and the *p*-values of Max.z(*l*) for them (0.0097, 0.01) were not good enough either. DINA was also rejected by M_2_ with a *p*-value below 0.05. DINO was narrowly accepted by M_2_ (*p*-value is 0.0799, close to 0.05). RMSEA and SRMSR gave favorable acceptance to all the CDMs, but it also indicated that the two indices might not be very sensitive about the model selection. G-DINA is a saturated model that can accommodate both compensatory and non-compensatory inter-attribute relationships, whereas A-CDM and LLM are compensatory models, and R-RUM is a non-compensatory one. A-CDM and R-RUM had the same absolute model fit at RMSEA (0 for both). These all indicated that both compensatory and non-compensatory models fit the data and the attributes of L2 listening, therefore, manifested both (non-)compensatory relationships. As DINA and DINO were rejected by Max.z(*r*) and Max.z(*l*), the inter-attribute relationships could not be interpreted simply as what either of the two models could accommodate.

**Table 3:** Absolute fit.

**CDMs**	**#par**	**Max.z(*r*)**	***p***	**Max.z(*l*)**	***p***	**M_**2**_**	***p***	**RMSEA**	**SRMSR**	**-2LL**	**AIC**	**BIC**
G-DINA	115	3.12	0.3046	2.89	0.6521	89.0495	0.128	0.0194	0.0431	11,259.24	11,489.23	11,973.91
DINA	101	4.68	0.0005	4.03	0.0097	118.336	0.0205	0.0257	0.0452	11,308.10	11,510.09	11,935.77
DINO	101	4.62	0.0006	4.02	0.0100	108.348	0.0799	0.0209	0.0209	11,300.98	11,502.98	11,928.65
A-CDM	108	3.34	0.1437	3.10	0.3272	75.9016	0.6686	0	0.0437	11,273.82	11,489.81	11,944.99
LLM	108	3.15	0.2831	2.96	0.5182	93.2377	0.1862	0.0166	0.0433	11,268.92	11,484.93	11,940.11
R-RUM	108	3.45	0.0947	3.21	0.2237	81.1406	0.5061	0	0.0441	11,278.34	11,494.35	11,949.52

*(a) #par., number of parameters; (b) Max.z(r) & Max.z(l), maximum z-score for transformed correlation and log odds ratio; (c) M_2_, a limited-information fit statistic for dichotomous response; (d) RMSEA, root mean square error of approximation; (e) SRMSR, standardized root mean square residual*.

Comparing among the three accepted reduced models (A-CDM, LLM, and R-RUM), LLM performed the best with the smallest−2LL (11268.92), AIC (11484.93), and BIC (11940.11). LLM had the smaller AIC (11484.93) and BIC (11940.11) than G-DINA (11489.23 and 11973.91, respectively). It does not mean that LLM is better than G-DINA, just that G-DINA invites larger penalties than LLM because AIC and BIC both introduce a penalty for model complexity. In this sense, LLM is the most comparable model with G-DINA at the test level.

### Model Selection at Item Level

As shown in Table 4, three models (DINO, DINA, and LLM) were selected; four items manifested compensatory inter-attribute relationships under DINO and LLM, whereas the other three items illustrated non-compensatory relationships under DINA. These results showed that the inter-attribute relationships tapped into by *L2LDA* were compensatory in some multi-attribute items and non-compensatory in others. Thus, the three models plus G-DINA formed Mixed-CDMs.

**Table 4:** Wald statistics for multi-attribute items.

	**Selected CDM**	**Wald statistics**	***p*-values**
Item 3	DINO	5.51	0.06
Item 4	DINA	3.57	0.17
Item 10	LLM	0.00	0.99
Item 11	DINO	1.16	0.56
Item 13	DINA	2.69	0.26
Item 16	DINA	0.17	0.92
Item 19	DINO	2.73	0.26

Table 5 shows that Mixed-CDMs had a high level of absolute fit, the significant levels of Max.z(*r*) and Max.z(*l*) were much higher than 1%, the *p*-value of M_2_ (0.3609) was much larger than 0.05, and RMSEA (0.0097) and SRMSR (0.0435) were below 0.05.

**Table 5:** Model fit of mixed-CDMs.

**#par**	**Max.z(*r*)**	***p***	**Max.z(*l*)**	***p***	**M_**2**_**	***p***	**RMSEA**	**SRMSR**	**-2LL**	**AIC**	**BIC**
102	2.78	0.9120	2.72	1.0000	92.1224	0.3609	0.0097	0.0435	11,274.36	11,478.36	11,908.25

### Comparisons Among G-DINA, LLM, and Mixed-CDMs

According to the results of the previous section, LLM was the most comparable model to G-DINA at the test level. Then Zoom-in comparisons were made among G-DINA, LLM, and Mixed-CDMs, and relative fit, absolute item fit, person fit, and the classification accuracy were concerned psychometric characteristics. As for the absolute fit at the test level, the three models all met the higher critical requirement (*p* > 0.10), but Mixed-CDMs were better than the other two with an increase from 0.3046 for G-DINA and 0.2831 for LLM to 0.9120 on Max.z(*r*) statistics and from 0.6521 and 0.5182 to 1 on Max.z(*l*) statistics (see Tables 3, 5). Moreover, the RMSEA of Mixed-CDMs was the smallest among the three. As for AIC, Mixed-CDMs performed the best on the test relative fit with the smallest value (11478.36), compared with G-DINA (11,489.23) and LLM (11,484.93). As shown in Table 6, the absolute item-level fit statistics for two items (Items 3 and 9) got improvement under Mixed-CDMs, whereas no statistically significant differences were noticed for other items. Mixed-CDMs improved the significant levels of Max.z(*r*) and Max.z(*l*) statistics from 3% for G-DINA and LLM to 10% and from 7% for G-DINA and 6% for LLM to 18%, respectively. Thus, Mixed-CDMs had better absolute item-level fit than G-DINA and LLM, especially on Items 3 and 9.

**Table 6:** Absolute item-level fit.

	**Item 3**				**Item 9**			
	**Max.z(*r*)**	***p***	**Max.z(*l*)**	***p***	**Max.z(*r*)**	***p***	**Max.z(*l*)**	***p***
G-DINA	3.12	0.03	2.89	0.07	3.12	0.03	2.89	0.07
LLM	3.14	0.03	2.92	0.06	3.14	0.03	2.92	0.06
Mixed-CDMs	2.79	0.10	2.58	0.18	2.79	0.10	2.58	0.18

Regarding person fit (Table 7), only one examinee (ID331) was over-fit (*l*_*z*_ > 2.0) under the three models. *l*_*z*_for ID331 under Mixed-CDMs was the smallest, and the mean absolute value of *l*_*z*_(|*l*_*z*_|) was also the smallest under Mixed-CDMs. Therefore, Mixed-CDMs were slightly better than G-DINA and LLM on person fit. In addition, the classification accuracy at the test level and attribute level were very close under Mixed-CDMs and G-DINA, which means that Mixed-CDMs were as reliable as G-DINA on the classification for the data.

**Table 7:** Psychometric characteristics under both models.

	**Mean of |*l_***z***_*| for person fit**	***l_***z***_* Index for ID331**	**Classification accuracy**							
			**Test-level**	**A1**	**A2**	**A3**	**A4**	**A5**	**A6**	**Means**
G-DINA	0.76	2.13	0.7176	0.8731	0.9119	0.9107	0.9253	0.9140	0.9152	0.9084
LLM	0.74	2.09	0.7954	0.8790	0.9931	0.9168	0.9274	0.9956	0.9197	0.9386
Mixed- CDMs	0.73	2.05	0.7157	0.8713	0.9158	0.9096	0.9083	0.9093	0.9138	0.9047

Item parameters of multi-attribute items are presented in Table 8. The standard errors were reduced under Mixed-CDMs in comparison with G-DINA and LLM. The estimates of item parameters under Mixed-CDMs were, therefore, statistically more accurate than those under G-DINA and LLM. G-DINA had the highest SE among the three CDMs.

**Table 8:** Item parameters estimates (EST) and standard errors (SE) of multi-attribute items.

**Items**	**Attributes**		**G-DINA**				**Mixed-CDMs**				**LLM**			
			**P(00)**	**P(10)**	**P(01)**	**P(11)**	**P(00)**	**P(10)**	**P(01)**	**P(11)**	**P(00)**	**P(10)**	**P(01)**	**P(11)**
Item 3	A2 + A4	EST	0.48	0.82	0.57	0.89	0.47	0.88	0.88	0.88	0.45	0.61	0.82	0.89
		SE	0.05	0.13	0.14	0.02	0.04	0.02	0.02	0.02	0.04	0.06	0.05	0.02
Item 4	A2 + A4	EST	0.34	0.17	0.57	0.71	0.35	0.35	0.35	0.74	0.33	0.31	0.71	0.69
		SE	0.04	0.15	0.14	0.03	0.03	0.03	0.03	0.03	0.04	0.05	0.05	0.03
Item 10	A4 + A5	EST	0.22	1.00	0.37	0.66	0.29	0.63	0.36	0.71	0.32	0.66	0.32	0.67
		SE	0.05	0.23	0.07	0.03	0.05	0.09	0.06	0.03	0.05	0.07	0.05	0.03
Item 11	A2 + A6	EST	0.23	0.79	1.00	0.89	0.26	0.90	0.90	0.90	0.31	0.38	0.89	0.92
		SE	0.04	0.10	0.26	0.03	0.04	0.02	0.02	0.02	0.04	0.06	0.04	0.02
Item 13	A4 + A5	EST	0.43	0.44	0.26	0.87	0.42	0.42	0.42	0.89	0.40	0.87	0.35	0.85
		SE	0.06	0.17	0.08	0.03	0.03	0.03	0.03	0.03	0.05	0.04	0.05	0.03
Item 16	A3 + A5	EST	0.34	0.29	0.35	0.82	0.34	0.34	0.34	0.84	0.28	0.73	0.37	0.80
		SE	0.06	0.13	0.06	0.03	0.03	0.03	0.03	0.03	0.04	0.06	0.04	0.03
Item 19	A4 + A5	EST	0.59	0.74	0.95	0.97	0.58	0.97	0.97	0.97	0.61	0.88	0.86	0.97
		SE	0.06	0.14	0.05	0.01	0.05	0.01	0.01	0.01	0.05	0.05	0.03	0.01

*EST, estimates; SE, standard error; P(11) refers to the probability of the right answer (PRA) to the item when two attributes are mastered; P(10) stands for the PRA when the first attribute is mastered; P(01) is the PRA when the second attribute is mastered*.

The item parameters also displayed the inter-attribute relationships *via* different PRAs (Table 8). If the PRA of an attribute was higher than 0.50, this attribute could compensate the other attribute with a more than 50% probability of giving the right answer. If the PRA of an attribute was below 0.50, this attribute could not compensate the other to give the right answer. Some common features were found under the three models. For Items 3 and 19, one attribute could compensate for the other under the three models. For Item 10, A4 could compensate A5 but not *vice versa*. So, the inter-attribute relationships exhibited compensatory traits in Items 3, 10, and 19 under the three models. In addition, compared with LLM, G-DINA shared more common features with Mixed-CDMs in terms of inter-attribute relationships. For Items 11, A2, and A6 could mutually compensate with each other under G-DINA and Mixed-CDMs, whereas A6 could compensate A2, not *vice versa*, under LLM. For Items 13 and 16, under both G-DINA and Mixed-CDMs, PRA for each attribute was below 0.50, which means that attributes could not compensate mutually for a right answer. Under LLM, however, compensatory traits were exhibited.

In addition to the inter-attribute relationships mentioned earlier, conflicting relationships were also detected in Table 8. For instance, under G-DINA, mastering both required subskills lowered the PRA (0.66) for Item 10 compared with mastering A4 only (PRA = 1). This sort of attribute conflict (mastering both attributes has lower PRA than mastering one attribute only) also existed in Item 11 under G-DINA and in Items 4 and 13 under LLM. However, this conflict was not exhibited under Mixed-CDMs.

### Content Analysis

The outcome of selecting models at the item level should be examined, whether theoretically valid or not, through content analysis. It deserved more attention when DINA and DINO were rejected at the test level but accepted at the item level. For better illustration, a special pair of attributes A2-A4 were focused because they were measured in two items: DINO was selected for one item, and DINA was for the other. Looking closely into the content of these items (Table 9), it was found that A2 (Vocabulary and Expressions) could compensate for the lack of A4 (Facts and Details) in Item 3, but A4 could not provide an equal probability of right answer if there were a lack of A2. Whether A4 could give a 50% probability of the right answer was partly because it was a multiple-choice item, and only two choices were logically pertinent. Therefore, DINO that allowed attribute to compensate for the lack of the other was not appropriate for this item, whereas G-DINA, which provided the real picture of the compensability: A2 could compensate for the lack of A4, but A4 could not compensate equally, should be retained. Item 4, in contrast with Item 3, required mastery of both A2 and A4. The expression “run out of” was one attribute (A2) measured in this item, but it did not provide a complete meaning unless it was combined with the detailed information “milk” (A4), which means when a test taker mastered both attributes, he/she could find the right answer. Thus, DINA was justified to be chosen for this item. In addition, mastery of the expression “run out of” (A2) offered lower PRA than mastery of none under G-DINA. This is unreasonable and uninterpretable, so G-DINA is not acceptable for this item. Therefore, item-level model selection still needs content analysis to detect flaws. The same pair of attributes may be able to exhibit different relationships: compensatory or non-compensatory, although DINO seemed to be improper for Item 3.

**Table 9:** Items tapping into attributes A2 and A4.

**Item**	**Attributes utilized**
3. M: Nancy, why are you late today? W: I overslept and missed the bus. Q: Why is Nancy late? a. The bus was late. b. Her clock was slow. c. She got up late. d. She forgot her class.	The item requires two attributes, A2 (Vocabulary and Expressions: overslept) and A4 (Facts and Details: missed the bus). A test taker would find answer key (c) if he/she only knows A2 (overslept), as it means the same as the answer key. However, if he/she only knows A4 (missed the bus), he/she would find (b) or (c), which means he/she has around 50% probability to find the right answer (c).
4. M: Where is Cindy? W: She ran out of milk and went to get some. Q: Why did Cindy go out? a. She went out jogging. b. She had no more milk. c. She went out for a walk. d. She was delivering milk.	A test taker would find answer key (b) only if he/she knows both the attributes, A2 (Vocabulary and Expressions: ran out of…) and A4 (Facts and Details: milk).

Following the same way, the rest two-attribute items were analyzed one by one. Interpretability is an important concern: if the inter-attribute relationship under a model is uninterpretable, then this model will be inferior, and the model that gives reasonable relationships will be accepted. In this way, it was found that the inter-attribute relationships under Mixed-CDMs are more reasonable and interpretable without conflicts, and then Mixed- CDMs were accepted for the rest two-attribute items.

## Discussions

The current study examined the selection of CDMs for an L2 listening comprehension test. Two-layer model selections mutually justified the mixed inter-attribute relationships of L2 listening subskills. The significance of this study is that statistically fit models at the item level require theoretical evidence informed by content analysis. The procedure used in this study can also serve as guidance for other studies aiming at choosing optimal CDM(s).

At the test level, A-CDM, LLM, and R-RUM were accepted and had a comparable relative fit with that of G-DINA. Based on the features of these CDMs, compensatory and non-compensatory inter-attribute relationships coexisted in the test, rather than a monotonous compensatory or non-compensatory one, which is different from previous findings (e.g., Yi, 2017). However, LLM and A-CDM have a smaller relative fit than R-RUM, which seemingly indicates that the inter-attribute relationships exhibit more compensatory than non-compensatory. However, how exactly the attributes interact with each other cannot be informed so far at the test level and would be overgeneralized under the assumed framework of a single reduced CDM if it is imposed on all items. Hence, multiple CDM selection at the item level within the same assessment is tenable and warranted.

At the item level, based on the selection criteria, DINA, DINO, and LLM were selected by Wald test for the seven multi-attribute items, G-DINA remained for 12 single-attribute items, and Mixed-CDMs were hence formed for the whole assessment by auto GDINA function. When the absolute fit, the relative fit, and the person fit were considered, Mixed-CDMs performed better over G-DINA and LLM, the most comparable with G-DINA. It is easy to understand that LLM is not optimal because it fails to postulate “both-and” relationships in its framework, even it is the most comparable with G-DINA. However, it is against the intuition that G-DINA is not superior to Mixed-CDMs for this dataset, although saturated G-DINA accommodates all the interactions among subskills and should have fit the data better. Sample size likely contributed to the result because saturated G-DINA needs a large sample size, and 500 was considered a small sample in previous studies (e.g., Chen et al., 2013; Ma et al., 2016). The small sample (*n* = 500) of this study might have constrained G-DINA's performance, which echoes the opinion that saturated models are not always the best choice when the sample size is small (Rojas et al., 2012; Ma et al., 2016). Based on the estimation of item parameters, standard errors of the estimates were the smallest under Mixed-CDMs, which suggests that the item parameter estimation under Mixed-CDMs was more accurate. These results largely agree with the claim of DiBello et al. (2007) that specific (or reduced) CDMs could reduce the standard errors when the sample size and the total number of items are small. It also renders empirical evidence to the simulation study of Ma et al. (2016) that “reduced CDMs usually require smaller sample sizes for accurate parameter estimation” (p. 201).

Although Mixed-CDMs were reported to be able to perform well-statistically, slightly better than G-DINA in some aspects, theoretical supports informed by the content analysis were needed. In this study, it was found that DINO selected by auto-GDINA function was not appropriate for Item 3 because the inter-attribute relationship manifested under the model could not reflect the underlying cognitive process of that item. G-DINA, however, could reflect this process and was thus retained for that item. Inexplicable conflicting inter-attribute relationships were found for some items under LLM and G-DINA (Table 8). No literature could interpret the conflicts. One plausible reason could be that LLM and G-DINA were not optimal models for the data and could not exhibit the real inter-attribute relationship. Content analysis of Item 4 showed that A2 (Vocabulary and expressions) and A4 (Facts and details) were non-compensatory with each other. DINA was accepted for this item, so inexplicable conflicting inter-attribute relationships occur under LLM and G-DINA. These sorts of conflicts under G-DINA were also mentioned in other studies (Meng, 2013; Chen and Chen, 2016; Ravand, 2016), and no linguistic explanation was provided because the interpretation of the conflicts was difficult based on the available language acquisition theories (Ravand, 2016), and they were considered as inherent inter-attribute relationships (Chen and Chen, 2016). Given that G-DINA and LLM provided inexplicable conflicts between subskills for some items, amended Mixed-CDMs are preferred because they better capture the inherent inter-attribute relationships of *L2LDA* and better reflect the processing of L2 listening without attribute conflicts.

After the amendment of the Mixed-CDMs by content analysis, four items selected compensatory models, whereas three items chose non-compensatory models. This provides evidence that compensatory and non-compensatory models coexist in the literature of listening tests (Buck and Tatsuoka, 1998; Sawaki et al., 2009; Yi, 2017). The items that chose compensatory models are slightly more than those that chose non-compensatory models. This could explain why, at the test level, compensatory LLM and A-CDM gained slight preference compared with non-compensatory R-RUM. This could also imply that test-level model acceptance could roughly predict inter-attribute relationships, and the accepted model having a better relative fit index could predict the dominant inter-attribute relationship. The small differences can also be accounted for by the small number of multi-attribute items (only seven items in this study), which has also been detected in the study of Lee and Sawaki (2009a) and the study of Yi (2017).

It is worth noting that two pairs of attributes were measured repetitively: A2–A4 and A4–A5. A2 and A4 were either compensatory or non-compensatory in two items of short conversations. In Item 4, only when “run out of” (A2) was combined with “milk” (A4), a complete semantic meaning could be understood. Therefore, A2–A4 was non-compensatory for this item. However, A2–A4 was compensatory in Item 3 because “overslept” (A2) and “missed the bus” (A4) each provided complete semantic meaning for understanding. As we can see, the semantic completeness of the target attributes may influence the attribute relationships for short conversations. The A4–A5 pair also showed flexible relationships, compensatory or non-compensatory. They were measured in a short conversation, a long conversation, and a video clip, respectively. In the short conversation, A4 (Facts and Details) was more important than A5 (Main Idea) and compensated A5. In the long conversation, A4 and A5 were not compensatory and had to work together to give the right answer. It seems that understanding facts and details (A4) is more important in processing less information (a short conversation) than understanding the main idea (A5), whereas understanding facts and details has to cooperate with understanding the main idea in processing more information (a long conversation). In the video item, A4 and A5 could compensate each other. It was not clear whether visual aid interacted with the two attributes in this item. The inconsistent inter-attribute relationships of L2 listening subskills are also congruent with the claim of Buck (2001) about varied patterns of relations and interactions of listening subskills. It depicted more detailed and varied relationships, which is in line with the finding of Yi (2017) on the aspect of rendering indirect evidence against the ability to define a hierarchy of contribution among listening subskills. The relationships vary from one item to another, and different features of items are likely to interact with subskills and influence their relationships. This interaction was referred to as “item–level interaction,” i.e., the same set of attributes may or may not exhibit interaction depending on the items that measure them (de la Torre et al., 2018). These findings imply that more consideration must be taken in test construction and validation. Compensatory relationships indicate that a correct answer could not guarantee every attribute in an item is actually used by the test taker.

Based on the discussion earlier, this study is significant in the following aspects:

First, it is found that test-level model acceptance by absolute fit indices can roughly predict inter-attribute relationships. This can also suggest whether item-level model selection should be needed or not. The model with a better relative fit index and an acceptable absolute fit index can predict the dominant inter-attribute relationship.
Second, the content analysis showed that the inexplicable attribute conflicts could be a signal of suboptimal model choice, and thus, item-level models are justified for better interpretations. The conflicts were also found in other studies (Meng, 2013; Chen and Chen, 2016; Ravand, 2016) but were not discussed and considered inherent inter-attribute relationships (Chen and Chen, 2016). Along this line, this study makes a step forward.
Third, the Mixed-CDMs are comparable with G-DINA for the L2 listening comprehension test and even better in some aspects. The amended Mixed-CDMs are optimal for *L2LDA*. Previous studies that involved the comparison between Mixed-CDMs and G-DINA were mainly based on simulations (Ma et al., 2016). This study provides a piece of useful empirical evidence for this topic and renders evidence to the study of L2 listening inter-subskill relationships.
Fourth, it is found that both compensatory and non-compensatory inter-subskill relationships exist in *L2LDA*, and even the relationships between the same pair of attributes are also non-fixed at different items. This is a new finding because the previous research reported only compensatory L2 listening inter-subskill relationships based on model selection (Yi, 2017). Semantic completeness of the attributes and item features are likely to interact with the subskills and influence the relationships. This was also rarely reported in previous studies, even less in L2 listening CDA research.
Last but not least, this study is significant in the procedure of model selection. Model selection is often seen in other assessment studies (Li et al., 2016; Ravand, 2016; Yi, 2017), but few on L2 listening assessment. In this study, the item-level model selection was initiated or ignited by the results of test-level model comparison and then was justified and amended by content analysis. The logic of the procedure provides useful insight into CDM studies.

## Limitations and Suggestions for Future Research

Although the findings of this research help provide a process of selecting the right CDM for L2 listening diagnostic assessment and shed some light on the inter-attribute relationships of L2 listening, part of the findings are possibly limited to the particular dataset and test used in this study.

*L2LDA* consisted of only 19 items. Although individual attributes were measured 4.15 times on average, one attribute was measured twice. According to Rupp et al. (2010), each attribute should be measured at least three times for accurate measurement with CDA.

Restricted by the test, only a few attribute pairs (i.e., A2–A4, A4–A5, A3–A5, and A2–A6) were measured. It may be inaccurate to judge inter-attribute relationships when the number of multi-attribute items is small. A3–A5 and A2–A6 were measured only once; this study did not opine what inter-attribute relationships they are. Thus, further research is needed to examine more pairs of attributes and more multi-attribute items.

Only one sample size (*n* = 500) was used in this study. Other small sample sizes (e.g., *n* = 300 or *n* = 600) should also be examined in future studies so that a feasible threshold of a small sample can be found to decide whether G-DINA or Mixed-CDMs should be used.

This study only focused on dichotomous items, so future studies are recommended to consider changing polychotomous items into binary coding or using some CDMs that accommodate polychotomous scales.

## Data Availability Statement

The raw data supporting the conclusions of this article will be made available by the authors, without undue reservation.

## Ethics Statement

The studies involving human participants were reviewed and approved by School of Foreign Studies of Xi'an Jiaotong University. The patients/participants provided their written informed consent to participate in this study.

## Author Contributions

YD: design of the study, data collection, data analysis, writing original draft, and revision. XM: supervision and writing-review. CW: supervision, writing-review, and editing. XG: data analysis. All authors contributed to the article and approved the submitted version.

## Conflict of Interest

The authors declare that the research was conducted in the absence of any commercial or financial relationships that could be construed as a potential conflict of interest.
